# *Campylobacter fetus* Invasive Infections and Risks for Death, France, 2000–2021

**DOI:** 10.3201/eid2911.230598

**Published:** 2023-11

**Authors:** Souheil Zayet, Timothée Klopfenstein, Vincent Gendrin, Jean-baptiste Vuillemenot, Julie Plantin, Lynda Toko, Nour Sreiri, Pierre-Yves Royer

**Affiliations:** Nord Franche-Comté Hospital, Trévenans, France

**Keywords:** *Campylobacter fetus*, bacteremia, secondary localizations, vascular, mortality, bacteria, France

## Abstract

*Campylobacter fetus* accounts for 1% of *Campylobacter* spp. infections, but prevalence of bacteremia and risk for death are high. To determine clinical features of *C. fetus* infections and risks for death, we conducted a retrospective observational study of all adult inpatients with a confirmed *C. fetus* infection in Nord Franche-Comté Hospital, Trevenans, France, during January 2000–December 2021. Among 991 patients with isolated *Campylobacter* spp. strains, we identified 39 (4%) with culture-positive *C. fetus* infections, of which 33 had complete records and underwent further analysis; 21 had documented bacteremia and 12 did not. Secondary localizations were reported for 7 (33%) patients with *C. fetus* bacteremia, of which 5 exhibited a predilection for vascular infections (including 3 with mycotic aneurysm). Another 7 (33%) patients with *C. fetus* bacteremia died within 30 days. Significant risk factors associated with death within 30 days were dyspnea, quick sequential organ failure assessment score >2 at admission, and septic shock.

*Campylobacter* is a genus of microaerophilic, fastidious, gram-negative, occasionally partially anaerobic, non–spore forming, motile bacteria with a characteristic spiral or corkscrew-like appearance (*1*). Such morphology enables the bacteria to colonize the mucosal surfaces of the gastrointestinal tract in humans and other animal species (*2*). In France, *C. fetus* is the most commonly isolated *Campylobacter* species, after *C. jejuni* and *C. coli*, found in fecal samples during diarrheal episodes in humans (*3*), and the leading species recovered from invasive infections, such as bacteremia and secondary localizations; both *C. jejuni* and *C. coli* have been identified in 43% of cases (*4*).

Earlier reports have revealed the incidence, clinical characteristics, and outcomes of bacteremia caused by *C. fetus* (*4*,*5*). Disease severity and risk for death from *C. fetus* systemic infection are of concern for clinicians; fatality rate is ≈15% (*4*,*5*). *C. fetus* is also known to have a predilection for vascular endothelium, causing mycotic aneurysms, thrombophlebitis, endocarditis (including infections of prosthetic heart valves), and multivisceral complications (*4*–*11*). A bactericidal antimicrobial drug treatment based on use of a β-lactam (such as amoxicillin/clavulanic acid or a carbapenem) should be favored (*4*).

Using data for January 2000–December 2021, we conducted a retrospective observational and descriptive study in Nord Franche-Comté Hospital, located in eastern France. Our primary objective was to describe clinical and paraclinical features (including antimicrobial susceptibility) in patients with *C. fetus* infections by comparing patients with and without bacteremia. Our secondary objective was to evaluate the risk factors for 30-day mortality in patients with bacteremia caused by *C. fetus.*

Patient consent was obtained by sending patients a letter informing them of the use of their medical data for research purposes and receiving no objection by 30 days later. Because of the retrospective nature of the study, with no patient involvement and use of already available data, the local Ethics Committee of Nord-Franche-Comte Hospital determined that patient consent was sufficient. The confidentiality of participant data has been respected in accordance with the Declaration of Helsinki.

## Methods

### Study Population and Design

Nord Franche-Comté Hospital has a capacity of 1,216 beds across all sites. The Nord-Franche-Comté Hospital practice has ≈100,000 visits to its emergency rooms and ≈3,600 deliveries per year (*12*,*13*). Our study included all adults (>18 years of age) with a *C. fetus* infection, defined by identification of *C. fetus* in a microbiological sample (blood, fecal, or other site culture) of hospitalized patients over a 21-year period (January 1, 2000–December 31, 2021).

### Data Collection

We collected clinical data regarding demographic and baseline characteristics and underlying conditions from patients’ medical records. We also extracted laboratory and imaging findings, outcomes, and results of antimicrobial susceptibility to amoxicillin, amoxicillin/clavulanic acid, imipenem, gentamicin, azithromycin, doxycycline, and fluoroquinolones (ofloxacin and ciprofloxacin).

### Definitions

We defined secondary localizations as a positive result on biopsy, graft, blood culture samples (or a combination of those) or evocative images on computed tomography or ^18^F-fluoro-deoxyglucose-positron emission tomography/computed tomography (^18^F-FDG PET/CT)**.** Endocarditis, also considered as a secondary localization, was defined by a positive valvular biopsy sample, blood culture, or both, associated with evocative images on echocardiography, ^18^F-FDG PET/CT, according to the European Society of Cardiology 2015 modified criteria for diagnosing infective endocarditis (*14*).

According to the Third International Consensus Definitions for Sepsis and Septic Shock (Sepsis-3) (*15*), adult patients with suspected infection can be rapidly identified as being more likely to have poor outcomes typical of sepsis if they have >2 of the following clinical criteria that together constitute a new bedside clinical score termed quick sequential (sepsis-related) organ failure assessment: respiratory rate of >22 minutes, altered mentation, or systolic blood pressure of <100 mm Hg.

### Microbiological Diagnosis

Until November 2016, strains were identified to the species level by biochemical tests associated with culture conditions and antimicrobial susceptibility and then by matrix-assisted laser desorption/time-of-flight ionization mass spectrometry methods (Microflex; Bruker Daltonics). Antimicrobial susceptibility was determined by using the disk-diffusion method; we reinterpreted susceptibility for this study according to guidelines of the Comité de l’Antibiogramme de la Société Française de Microbiologie (CA-SFM)/European Committee on Antimicrobial Susceptibility Testing (EUCAST) 2021 version 1. CA-SFM/EUCAST is a version of EUCAST translated into French and adjusted for practices in France (*16*). We considered antimicrobial treatment to be appropriate if the strain was susceptible to >1 of the drugs prescribed, according to the CA-SFM/EUCAST recommendations (*17*). *C. fetus* is naturally resistant to third-generation cephalosporins, ticarcillin, and piperacillin, so we considered those drugs to be inappropriate. Some strains were tested by automated broth microdilution system (Vitek-2; bioMérieux), and results could not be reinterpreted according to current recommendations.

### Data Analysis

Unless otherwise indicated, we expressed discrete variables as numbers and percentages and continuous variables as mean/average, SD, and 95% CI**.** We performed comparisons among patients with and without *C. fetus* bacteremia by using a χ^2^ or Fisher exact test for qualitative variables and a Student *t* or Wilcoxon test for quantitative data. Risk factors for death are expressed as odds ratios (ORs), and statistical analysis was performed by using univariate logistic regression. We used a significance level of p>0.05 and performed all analyses by using R version 4.2.1 (The R Project for Statistical Computing, https://www.r-project.org). We defined a significant trend as p<0.06.

## Results

We considered conventional methods to be the standard combined with matrix-assisted laser desorption/time-of-flight ionization mass spectrometry identification, which enabled us to identify 991 *Campylobacter* species. The main species found was *C. jejuni* (823 [83%]), followed by *C. coli* (70 [7%]). The third most frequently found species was *C. fetus* (39 [4%]) and the fourth was *C. upsaliensis* (12 [1%]). *Campylobacter* species were not identified in 47 (5%) isolates.

**Figure 1:**
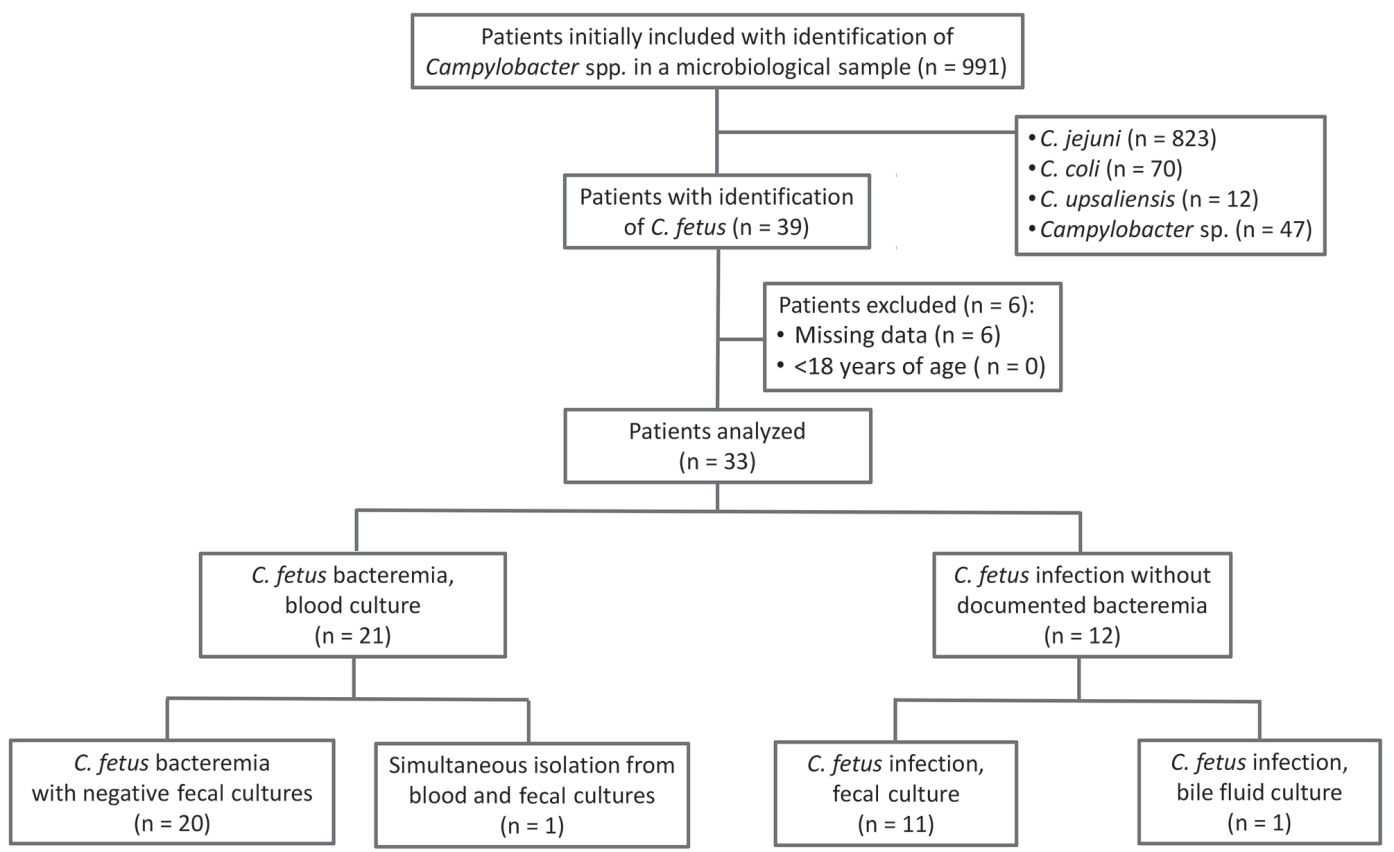
Flowchart of patient enrollment in study of *Campylobacter fetus* invasive infections and risks for death, Nord Franche-Comté Hospital, France, 2000–2021.

During the study period, we identified 39 patients with culture-positive *C. fetus* infections; of those, 33 had complete records and underwent further analysis, 21 with documented bacteremia and 12 without (Figure 1). Among bacteremic patients, fecal cultures were negative for 20 (95.3%). For only 1 patient were simultaneous peripheral blood and stool cultures positive, isolating *C. fetus*, and that patient was included in the bacteremia group. With regard to patients without documented bacteremia, most (11 of 12) isolates were from fecal samples; gastroenteritis was reported for 10 (83%) patients, of which 9 had liquid diarrhea. Peripheral blood cultures were performed for 8 (66%) of 12 patients without documented *C. fetus* bacteremia and were negative.

### Demographic and Epidemiologic Data

**Figure 2:**
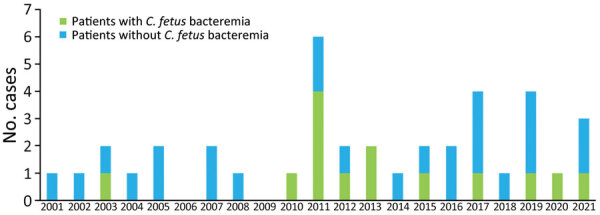
Distribution over time of bacteremia in 33 patients with *Campylobacter fetus* infection, Nord Franche-Comté Hospital, France, 2000–2021.

The mean prevalence of *C. fetus* infection was 1.5 cases/year. During the study, the highest incidence rate was noted in 2011 (6 [18%] cases) (Figure 2). The mean age of the study population was 73 (SD 18) years, and male patients (54%) were predominant. Patients with *C. fetus* bacteremia were older than patients without bacteremia, but the difference was not significant (77 [SD 16] vs. 66 [SD 19] years; p = 0.12). Immunosuppression was more frequent in patients with *C. fetus* bacteremia, and the trend was significant (52% [11/21] vs. 16% [2/12]; p = 0.06); malignancy/cancers were the leading cause (7/21 [33%]). Among the 21 patients with bacteremia, the main underlying conditions were cardiovascular disease (15 [71%]), diabetes mellitus (7 [33%]), renal failure (6 [29%]), and prosthetic heart valves (4 [19%]); no significant difference compared with patients without documented *C. fetus* bacteremia was noted. Not represented by our study population were pregnancy, contact with livestock, poultry consumption, and similar cases in the household (Table 1).

**Table 1:** Baseline characteristics, clinical features, and laboratory, and imaging findings for patients with *Campylobacter fetus* infections, Nord Franche-Comté Hospital, Trévenans, France, 2000–2021*

Characteristics	All patients, n = 33	Patients without documented *C. fetus* bacteremia, n = 12†	Patients with documented *C. fetus* bacteremia, n = 21	p value
Baseline				
Sex				0.7
M	18/33 (54)	6/12 (50)	12/21 (57)	
F	15/33 (45)	6/12 (50)	9/21 (43)	
Age, y (SD)	73 (±18)	66 (±19)	77 (±16)	0.12
Underlying comorbidities	27/33 (82)	8/12 (67)	19/21 (90)	0.2
Immunosuppression	**13/33 (39)**	**2/12 (16)**	**11/21 (52)**	**0.06**
Malignancy/cancers	8/33 (24)	1/12 (8.3)	7/21 (33)	0.2
Transplantation	0/33	0/12	0/21	
Cirrhosis	1/33 (3.0)	0/12	1/21 (4.8)	>0.9
HIV infection	0/33	0/12	0/21	
Connective tissue disease‡	3/33 (9.0)	1/12 (8.3)	2/21 (9.5)	>0.9
Other§	1/33 (3.0)	0/12 (8.3)	1/21 (29)	>0.9
Prosthetic heart valves	8/33 (24)	4/12 (33)	4/21 (19)	0.4
Renal failure	7/33 (21)	1/12 (8.3)	6/21 (29)	0.2
Diabetes mellitus	12/33 (36)	5/12 (42)	7/21 (33)	0.7
Cardiovascular disease¶	20/33 (61)	5/12 (42)	15/21 (71)	0.14
Recent abdominal surgery	1/33 (3.0)	0/12	1/21 (4.8)	>0.9
Pregnancy	0/33	0/12	0/21	
Contact with livestock/cattle	0/31	0/11	0/20	
Poultry consumption	0/31	0/11	0/20	
Recent travel#	1/31 (3.2)	1/11 (9.1)	0/20	0.4
Similar cases in household/entourage	0/31	0/11	0/20	
Recent abdominal trauma injury	4/31 (13)	2/11 (18)	2/20 (10)	0.6
Transmission or contamination, food or pet	2/31 (6.5)	0/11	2/20 (10)	0.5
Clinical features				
Fever	21/32 (66)	8/11 (73)	13/21 (62)	0.7
Headache	1/32 (3.1)	0/11	1/21 (4.8)	>0.9
Asthenia/anorexia	16/33 (48)	4/12 (33)	12/21 (57)	0.3
Abdominal pain	**11/33 (33)**	**7/12 (58)**	**4/21 (19)**	**0.052**
Confusion	8/31 (26)	2/10 (20)	6/21 (29)	>0.9
Dyspnea, respiratory rate >22 cycles/min	8/31 (26)	1/10 (10)	7/21 (33)	0.2
Hypotension, systolic pressure <100 mm Hg	7/31 (23)	1/10 (10)	6/21 (29)	0.4
qSOFA score				0.4
0	20/31 (65)	8/10 (80)	12/21 (57)	
1	3/31 (10)	1/10 (10)	2/21 (10)	
2	6/31 (19)	0/10	6/21 (29)	
3	2/31 (6.5)	1/10 (10)	1/21 (4.8)	
Sepsis: qSOFA ≥2	8/31 (26)	1/10 (10)	7/21 (33)	0.22
Diarrhea, n = 33				
Total	**16/33 (48)**	**10/12 (83)**	**6/21 (29)**	**0.004**
Liquid	14/33 (42)	9/12 (75)	5/21 (24)	**0.009**
Bloody	2/33 (6.1)	1/12 (8.3)	1/21 (4.8)	>0.9
Laboratory and imaging findings				
Leukocyte count, cells/mm^3^ (SD); reference range 4,000–10,000 cells/mm^3^	12,387 (6,665)	9,949 (3,307)	13,548 (7,571)	0.076
CRP, mg/L (SD), reference range <5 mg/L	118 (79)	120 (52)	116 (93)	0.9
Peripheral blood culture				
No. (SD)	NA	NA	2.52 (1.63)	NA
Negativation, d (SD)	NA	NA	2.89 (3.14)	NA
Transthoracic echocardiography**	4/32 (12)	1/11 (9.1)	3/21 (14)	>0.9
^18^F-FDG PET/CT	3/32 (9.4)	0/11 (0)	3/21 (14)	0.5

*Values are no./total (%) except as indicated. Boldface indicates p<0.05 or a significant trend defined by p<0.06. Blank cells for p values indicate no p value was calculated. ^18^F-FDG PET/CT, ^18^F-fluoro-deoxyglucose-positron emission tomography/computed tomography; CRP, C-reactive protein; qSOFA, quick sequential organ failure assessment; NA, not applicable. †*Campylobacter fetus* was isolated from 11 fecal cultures and from 1 bile fluid culture; only 1 patient had simultaneous positive peripheral blood and fecal cultures in which *C. fetus* was isolated with the same antimicrobial testing susceptibility pattern. ‡All patients with connective tissue diseases (bacteremia; n = 2) had multiple sclerosis. §Defined by hematologic diseases, long-term steroid therapy, or immunomodulatory treatment. ¶Defined by cardiac failure, arrhythmia, coronary heart disease, stroke, peripheral arterial obstructive disease and thromboembolic disease. #28-y-old patient sought care for bloody diarrhea without fever 8 d after a travel in Spain. **The 3 patients who underwent transthoracic echocardiography were different patients than those who underwent ^18^F-FDG PET/CT. No patient had both endocarditis and mycotic aneurysm.

### Clinical Features, Laboratory Data, and Imaging Findings

The predominant clinical sign was fever; no significant difference was found between the 2 groups (62% [13/21] vs. 73% [8/11]; p = 0.7). Gastrointestinal signs/symptoms were more common among patients with no bacteriemia (abdominal pain, 58% vs. 19%; p = 0.052; diarrhea, 75% vs. 24%; p = 0.009); the difference was significant. At admission, average leukocyte count was higher for patients with *C. fetus* bacteremia (13,550 [SD 7.57] cells/mm^3^) than without bacteremia (9,950 [SD 3.31] cells/mm^3^); the difference was not significant (p = 0.076). C-reactive protein level was equivalent in both groups (116 [SD 93] mg/L vs. 120 [±52] mg/L; p = 0.9). Transthoracic echocardiography (TTE) and ^18^F-FDG PET/CT were performed for 3 bacteremic patients. TTE indicated 1 case of prosthetic valve endocarditis and revealed typical oscillating vegetation. ^18^F-FDG PET/CT confirmed the diagnosis of mycotic aneurysm in 3 other patients (Table 1).

### Secondary Localizations

Secondary localizations were exclusively observed in one third of patients with *C. fetus* bacteremia (7/21 [33%]; p = 0.03). A predilection for vascular infections was noted for 5 patients (3 with mycotic aneurysm, 2 with percutaneous implantable port-related infection/thrombophlebitis), and 1 had endocarditis, and 1 had septic arthritis (Table 2).

**Table 2:** Secondary localizations, therapeutic management, and outcomes of patients with *Campylobacter fetus* infections among patients with and without bacteremia, Nord Franche-Comté Hospital, Trévenans, France, 2000–2021*

Characteristics		All patients, n = 33	Patients with no *C. fetus* bacteremia, n = 12	Patients with *C. fetus* bacteremia, n = 21	p value
Secondary localizations†					
Site infection					
Total		**7/33 (21)**	**0/12 **	**7/21 (33)**	**0.03**
Mycotic aneurysm		3/33 (9.0)	0/12	3/21 (14.3)	0.28
Endocarditis		1/33 (3.0)	0/12	1/21 (4.8)	>0.9
Infection associated with a medical device		1/33 (3.0)	0/12	1/21 (4.8)	>0.9
Thrombophlebitis		1/33 (3.0)	0/12	1/21 (4.8)	>0.9
Bone or joint infection		1/33 (3.0)	0/12	1/21 (4.8)	>0.9
Skin or soft tissue/abscesses		0/33 (0)	0/12	0/21 (0)	NA
Meningitis		0/33 (0)	0/12	0/21 (0)	NA
Antimicrobial therapy					0.6
Amoxicillin		0/29 (0)	0/9	0/20 (0)	
Amoxicillin–clavulanic acid		12/29	2/9	10/20 (50)	
Imipenem		2/29 (6.9)	0/9	2/20 (10)	
Gentamicin		3/29 (10)	0/9)	3/20 (15)	
Azithromycin		1/29 (3.4)	1/9 (11)	0/20 (0)	
Ciprofloxacin		3/29 (10)	2/9 (22)	1/20 (5.0)	
Other		11/29 (38)	3/9 (33)	8/20 (40)	
No. antimicrobial drugs/patient					0.065
0		4/29 (17)	2/9 (22)	2/20 (10)	
1		11/29 (38)	5/9 (56)	6/20 (30)	
2		10/29 (34)	2/9 (22)	8/20 (40)	
3		4/29 (14)	0/9	4/20 (20)	
Dual-therapy regimens					
Amoxicillin/clavulanic acid + gentamicin		3/10 (30)	0/2	3/8 (38)	
Amoxicillin/clavulanic acid + azithromycin		1/10 (10)	0/2	1/8 (12)	
Amoxicillin/clavulanic acid + ciprofloxacin		2/10 (20)	0/2	2/8 (25)	
Amoxicillin/clavulanic acid + doxycycline		3/10 (30)	2/2 (100)	1/8 (12)	
Imipenem + gentamicin		1/10 (10)	0/2	1/8 (12)	
Treatment duration, d (SD)		8 (8)	5 (5)	9 (8)	0.2
Outcomes and mortality rates					
Long-term complications, n = 33					
Total		1/33 (3.0)	0/12	1/21 (4.8)	>0.9
Aneurysmal rupture/aortic dissection		1/33 (3.0)	0/12	1/21 (4.8)	>0.9
Acute coronary syndrome		0/33 (0)	0/12	0/21 (0)	NA
Irritable bowel syndrome		0/33 (0)	0/12	0/21 (0)	NA
GBS (polyradiculoneuritis)		0/33 (0)	0/12	0/21 (0)	NA
Surgery‡		5/31 (16.1)	1/10	4/21 (19)	0.6
Relapse§		2/33 (6)	0/12	2/21 (9.5)	0.5
Transfer to intensive care		4/33 (12)	0/12	4/21 (19)	0.3
Septic shock		4/33 (12)	0/11	4/21 (19)	0.3
Infection-related mortality		6/29 (21)	1/8 (12)	5/21 (24)	0.6
30-day mortality rate¶		10/33 (30)	3/12 (25)	7/21 (33)	0.9

*Values are no./total (%) except as indicated. Boldface indicates p<0.05 or a significant trend defined by p<0.06. Blank cells for p values indicate no p value was calculated. GBS, Guillain-Barré syndrome; NA, not applicable. †Mycotic aneurysm (n = 3) with infectious native aortic aneurysm (n = 2); prosthetic aortic valve and a positive culture of the aneurysm after surgery (n = 1); prosthetic valve endocarditis (n = 1) with typical oscillating vegetation (15 mm) confirmed by transthoracic echocardiography; abdominal aorta thrombophlebitis (n = 1); hematogenous medical device infection with a percutaneous implantable port-related infection (n = 1); osteoarticular (n = 1) with glenohumeral shoulder arthritis and a positive culture of the articular fluid after surgery, suggesting a contiguous infection. ‡Four patients with bacteremia caused by *C. fetus* underwent surgery: mycotic aneurysm (n = 2), endocarditis (n = 1), and septic arthritis (n = 1). §Two patients exhibited a relapse with fever after 26 and 50 d; the second patient died of septic shock during the second episode. The first patient received ciprofloxacin orally for 5 d, and the second patient received IV vancomycin for 7 d. ¶Among the 7 bacteremic patients who died, 2 died in the context of evolutive/expanding malignancy (independently of the bacteremia).

### Therapeutic Management and Outcomes

Among 29 patients receiving antimicrobial therapy, the most commonly used drug was amoxicillin/clavulanic acid (12 [41%]). The most common choice for treating *C. fetus* bacteremia was dual-regimen therapy (8/20 [40%]). Amoxicillin/clavulanic acid was prescribed for 7 (88%) of 8 patients with bacteremia treated with dual-regimen therapy. We found no significant difference in mean duration of treatment between the 2 groups (9 [SD 8] vs. 5 [SD 5] days; p = 0.2). Five patients underwent surgery, including 4 with bacteremia (2 for mycotic aneurysm, 1 for prosthetic valve endocarditis, and 1 for septic arthritis). Four patients were transferred to an intensive care unit for septic shock. Two patients experienced a relapse with fever as the main clinical sign after 26 and 50 days; 1 patient died of septic shock during the second episode.

### Antimicrobial Susceptibility Testing

Among patients with *C. fetus* bacteremia, the rate of resistance was 10% (2/20) to both amoxicillin and azithromycin and 33% (6/18) to fluoroquinolones. No resistance to amoxicillin/clavulanic acid, imipenem, and gentamicin was noted (Table 3).

**Table 3:** Antimicrobial resistance of *Campylobacter fetus* strains isolated from patients with and without bacteremia, Nord Franche-Comté Hospital, Trévenans, France, 2000–2021*

Antimicrobial tested	All patients, no. (%), n = 33	Patients with no *C. fetus* bacteremia, no. (%), n = 12	Patients with *C. fetus* bacteremia, no. (%), n = 21	p value
Amoxicillin†	3/28 (10.7)	1/8 (12)	2/20 (10)	0.3
Amoxicillin–clavulanic acid	0/28	0/8	0/20	
Imipenem	0/28	0/8	0/20	
Gentamicin	0/27	0/8	0/19	
Azithromycin	2/29 (6.9)	0/9	2/20 (10)	>0.9
Fluoroquinolones: ofloxacin and ciprofloxacin‡	8/27 (30)	2/9 (22)	6/18 (33)	0.7
Doxycycline	7/29 (24)	1/9 (11)	6/20 (30)	0.4

*Values are no. resistant/no. tested (%) except as indicated. Blank cells for p values indicate no p value was calculated. †Susceptibility to amoxicillin was intermediate (susceptible with high doses) for 2 strains. ‡Susceptibility to ofloxacin and ciprofloxacin was the same for all strains.

### Mortality Rate

**Figure 3:**
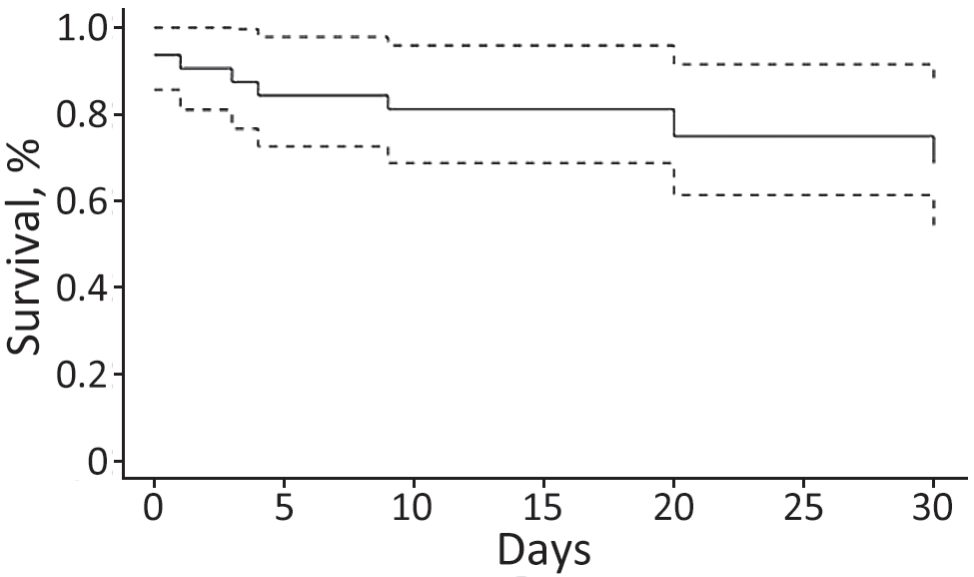
Kaplan-Meier survival curve for 33 patients with *Campylobacter fetus* infection, with and without bacteremia, Nord Franche-Comté Hospital, France, 2000–2021. Dashed lines indicate 95% CIs.

The global 30-day mortality rate was estimated at 30% (10/33); no significant difference between the 2 groups was noted (33% [7/21] vs. 25% [3/12]; p = 0.9) (Figure 3). Seven (33%) patients with *C. fetus* bacteremia died within 30 days. Among them, 2 patients died of evolutive/expanding neoplasia, independent of the *C. fetus* bacteremia. Significant risk factors associated with the 30-day mortality rate were dyspnea (OR 15.0, 95% CI 1.9–186.4; p = 0.017), quick sequential organ failure assessment score at admission >2 (OR 4.9, 95% CI 1.6–21.9; p = 0.012), and septic shock (OR not applicable; p = 0.006). Protective factors were initial prescription of amoxicillin–clavulanic acid (OR 0.09; 95% CI 0–0.75; p = 0.05) and use of dual antimicrobial therapy (OR not applicable; p = 0.001) (Table 4).

**Table 4:** Risk factors for death within 30 d after *Campylobacter fetus* bacteremia, Nord Franche-Comté Hospital, Trévenans, France, 2000–2021*

Variable	Total, n = 21	Survival, n = 14	Death, n = 7	p value†	OR (95% CI)
Dyspnea, respiratory rate >22 cycles/min	7/21 (33)	2/14 (14)	5/7 (71)	0.017	15.0 (1.9–186.4)
qSOFA score				0.017	NA
0	12/21 (57)	11/14 (79)	1/7 (14)		
1	2/21 (10)	1/14 (7.1)	1/7 (14)		
2	6/21 (24)	2/14 (14)	4/7 (57)		
3	1/21 (4.8)	0/14	1/7 (14)		
qSOFA score				0.012	4.9 (1.6–21.9)
Sepsis: qSOFA score >2	7/21 (33)	2/14 (14)	5/7 (71)	0.021	13.7 (1.7–171.4)
Septic shock	4/21 (19)	0/14	4/7 (57)	0.006	NA
Transfer to intensive care	4/21 (19)	0/14	4/7 (57)	0.006	NA
Treated with amoxicillin/clavulanic acid	10/21 (48)	9/14 (64)	1/7 (14)	0.05	0.09 (0.0–0.75)
Antimicrobial drugs/patient				0.001	NA
0	3/21 (14)	0/14	3/7 (43)		
1	6/21 (29)	5/14 (36)	1/7 (14)		
2	8/21 (38)	8/14 (57)	0/7		
3	4/21 (19)	1/14 (7.1)	3/7 (43)		
Antimicrobial drugs/patient, median	1.6	1.7	1.4	0.519	0.7 (0.2–1.9)
Infection-related mortality	5/21 (24)	0/14	5/7 (71)	0.001	NA

*Values are no./total (%) except as indicated. Blank cells for p values indicate no p value was calculated. qSOFA, quick sequential organ failure assessment; NA, not applicable; OR, odds ratio. †p<0.05 indicates significance.

## Discussion

The most commonly detected cause of *Campylobacter* bacteremia is *C. fetus* (*5*,*6*). However, cohorts or large case series exclusively involving patients with *C. fetus* bacteremia remain scarce. *C. fetus* is usually isolated from blood samples and is less frequently associated with enteritis (*18*,*19*). The Campylobacteremia Study (*4*), a retrospective multicentric study of *Campylobacter* spp. bacteremia in France, also showed that one of the regions with the highest rate of *Campylobacter* spp. infection is the Franche-Comté region. 

Our study comprised 33 patients with *C. fetus* infection; the 21 patients with bacteremia were older than the patients without bacteremia, in keeping with data in the literature and previous reports. According to the medical literature of patients with *Campylobacter* bacteremia in one of the largest retrospective cohorts (n = 592), patients were elderly (median age 68 years) and most had underlying conditions, mainly immunosuppression (*4*). In our cohort, immunosuppression was more frequent among patients with bacteremia caused by *C. fetus* than among with patients with no bacteremia; the trend was significant (p = 0.06). The leading cause was malignancy or cancer (33%) (*1*,*20*). Two patients with documented *C. fetus* bacteremia had systemic sclerosis, which seems to be a predisposing condition among connective tissue diseases (*1*,*21*). Pacanowski et al. (*5*) showed that, compared with patients with bacteremia caused by other *Campylobacter* species, patients with *C. fetus* bacteremia were older and had underlying comorbidities (e.g., cardiovascular diseases, diabetes mellitus). That finding is consistent with our results and those of other reports (*20*,*22*).

Among patients with *C. fetus* bacteremia, one third exhibited secondary localizations with a predilection for vascular infections. A recent multicenter study in France (252 patients with *C. fetus* bacteremia) found that 11.5% patients had vascular localization and 4.4% had endocarditis (*6*). In our study population, we found more vascular localizations but less endocarditis. However, secondary endovascular localizations were not systematically searched and might have been underdiagnosed.

In our study, TTE and ^18^F-FDG PET/CT were each performed for only 14% of patients with bacteremia, which is a major limitation. We suggest performing those radiologic examinations early for patients with *C. fetus* bacteremia (*6*,*23*). Late radiologic examination may partially explain the high mortality rate among patients with aneurysm rupture or endocarditis.

One of the major problems associated with *C. fetus* infection is empiric treatment. Infection with those fastidious bacteria is uncommon, and recommendations for treatment of bacteremia are lacking. The standard choice for empiric treatment of *Campylobacter* spp. enteritis remains fluoroquinolones and macrolides (*18*). However, in our cohort, 33% of bloodstream isolates were resistant to fluoroquinolones, and 10% were resistant to azithromycin. No strain was resistant to amoxicillin/clavulanic acid, aminoglycoside, or imipenem. The initial empiric treatment should be dual antimicrobial therapy (including amoxicillin/clavulanic acid or imipenem with an aminoglycoside) (*6*,*22*,*24*). In our cohort, all dual-therapy regimens consisted of amoxicillin/clavulanic acid (7/8 [88%]) or imipenem (1/8 [12%]) with a second agent. We conclude that initial prescription of amoxicillin/clavulanic acid and use of dual antimicrobial therapy were protective factors. Failure to administer appropriate antimicrobial therapy is strongly associated with fatal outcomes (*4*,*5*). 

Other independent risk factors for death were immunosuppression, cancers, and surgery (*5*,*6*). In our cohort, risk factors for death within 30 days after *C. fetus* bacteremia were dyspnea, quick sequential organ failure assessment score at admission >2, and septic shock. We found no significant difference between survivors and nonsurvivors with regard to antimicrobial therapy duration (p = 0.8), which could be explained by the longstanding clinician behavior of avoiding short antimicrobial regimens, even for patients who have positive fecal cultures without bacteremia.

In our cohort, the mortality rate was high (33% of patients with *C. fetus* bacteremia). It should be noted that among those 7 patients, 2 died in the context of evolutive/expanding malignancy and 1 died in the context of recurrent bacteremia with septic shock. In addition, 3 of 7 bacteremic patients who died were receiving 3 antimicrobial drugs, which suggests that in some cases, the number of antimicrobial drugs may have been a marker of illness severity.

Among the limitations of our study were the retrospective method used and the limited number of patients. A prospective study might confirm and support our results. As we previously mentioned, secondary localizations are probably underdiagnosed because of lack of knowledge of this disease and therefore nonperformance of investigations.

In summary, we found that *C. fetus* bacteremia mainly affects patients who are elderly, are immunocompromised, or have underlying conditions. Infections are associated with high mortality rates, especially if no dual antimicrobial therapy including amoxicillin/clavulanic acid is prescribed. For patients with bacteremia caused by *C. fetus*, screening for secondary localizations may be warranted by performing TTE and ^18^F-FDG PET/CT.
